# Supplementary material for: Urea fertilization and grass species alter microbial nitrogen cycling capacity and activity in a C4 native grassland
Source: PeerJ. 2022 Aug 12;10:e13874. doi: 10.7717/peerj.13874 (PMC9377331; doi:10.7717/peerj.13874)
Supplement: Supplemental Information 2 — From Hu et al. (2021a). [file peerj-10-13874-s002.docx]

**Table S1.** **Primer sets sequences and thermocycling conditions used for qPCR and qRT-PCR**. From Hu et al., 2021a.

| Gene | Primers | Sequence (5'-3') | Reaction parameters |
| --- | --- | --- | --- |
| *amoA* (AOB^†^) | *amoA* 1F  *amoA* 2R | GGGGTTTCTACTGGTGGT  CCCCTCKGSAAAGCCTTCTTC | 95 ℃ for 3 min × 1 cycle; (94 ℃ for 1 min, 56 ℃ for 45 s, 72 ℃ for 1 min) × 40 cycles; 72 ℃ for 10 min × 1 cycle |
| *nifH* | IGK3  DVV | GCIWTHTAYGGIAARGGIGGIATHGGIAA  ATIGCRAAICCICCRCAIACIACRTC | 95 ℃ for 10 min × 1 cycle; (95 ℃ for 30 s, 58 ℃ for 1 min, 72 ℃ for 1 min) × 40 cycles; 72 ℃ for 10 min × 1 cycle |
| *nirS* | cd3aF  R3cd | GTSAACGTSAAGGARACSGG  GASTTCGGRTGSGTCTTGA | 95 ℃ for 10 min × 1 cycle; (94 ℃ for 30 s, 57 ℃ for 1 min, 72 ℃ for 1 min) × 40 cycles; 72 ℃ for 10 min × 1 cycle |
| *nirK* | F1aCu  R3Cu | ATCATGGTSCTGCCGCG  GCCTCGATCAGRTTGTGGTT | 94 ℃ for 2 min × 1 cycle; (94 ℃ for 30 s, 58 ℃ for 1 min, 72 ℃ for 1 min) × 40 cycles; 72 ℃ for 10 min × 1 cycle |
| *nosZ* | *nosZ*-I F  *nosZ*-I R | CGCRACGGCAASAAGGTSMSSGT  CAKRTGCAKSGCRTGGCAGAA | 94 ℃ for 5 min × 1 cycle; (94 ℃ for 40 s, 60 ℃ for 40 s, 72 ℃ for 1 min) × 40 cycles; 72 ℃ for 10 min × 1 cycle |

^†^AOB, ammonia-oxidizing bacteria

Hu, J., Jin, V. L., Konkel, J. Y. M., Schaeffer, S. M., Schneider, L. G., & DeBruyn, J. M. (2021a). Soil health management enhances microbial nitrogen cycling capacity and activity. mSphere, 6(1), e01237-01220. https://doi.org/10.1128/mSphere.01237-20
