# Supplementary material for: Urea fertilization and grass species alter microbial nitrogen cycling capacity and activity in a C4 native grassland
Source: PeerJ. 2022 Aug 12;10:e13874. doi: 10.7717/peerj.13874 (PMC9377331; doi:10.7717/peerj.13874)
Supplement: Supplemental Information 3 — Different lowercase letters indicate significant differences between treatment levels within groups. The letters were only shown in groups that have significant effects. Means were compared using Least Significant Difference (LSD) tests (α = 0.05). Data are from Hu et al. (2021b). [file peerj-10-13874-s003.docx]

**Table S1**. **Soil properties in relation to agricultural season, N fertilization rate, and grass species.** Different lowercase letters indicate significant differences between treatment levels within groups. The letters were only shown in groups that have significant effects. Means were compared using Least Significant Difference (LSD) tests (α = 0.05). Data are from Hu et al. (2021b).

| Factor^†^ | pH | SWC^‡^  (%) | NH_4_^+^-N | NO_3_^-^-N | DOC | DON | Total C | Total N | C: N ratio | N_2_O-N  (g ha^-1^ d^-1^) | NP (μg NO_2_-N gdw^-1^ hr^-1^) |
| --- | --- | --- | --- | --- | --- | --- | --- | --- | --- | --- | --- |
|  |  |  | (μg gdw^-1^) | | | | (mg gdw^-1^) | |  |  |  |
| April (G) | 6.27±0.05 | 24.46±0.25^b*^ | 10.82±0.49^a^ | 1.51±0.30^a^ | 203.55±4.20^b^ | 18.67±0.56^ab^ | 1.82±0.03 | 21.74±0.43 | 11.94±0.11 | 9.71±0.94^b^ | 0.06±0.01^c^ |
| June (H1) | 6.30±0.06 | 30.16±0.46^a^ | 10.57±0.60^a^ | 2.14±0.46^a^ | 163.85±4.07^c^ | 16.16±0.65^b^ | 1.76±0.06 | 21.18±0.68 | 12.05±0.15 | 24.77±7.44^a^ | 0.11±0.02^b^ |
| August (H2) | 6.18±0.03 | 15.03±0.40^c^ | 7.16±0.35^b^ | 0.83±0.25^b^ | 223.00±5.47^a^ | 22.44±1.98^a^ | 1.84±0.05 | 21.66±0.51 | 11.81±0.12 | 1.64±0.17^b^ | 0.18±0.02^a^ |
| 0N | 6.36±0.05^a^ | 23.27±1.63 | 9.37±0.85 | 0.39±0.16^c^ | 210.18±8.60^a^ | 17.65±1.10 | 1.71±0.04 | 20.73±0.53 | 12.11±0.16 | 7.55±1.49^b^ | 0.06±0.01^c^ |
| 67N | 6.29±0.04^a^ | 23.39±1.66 | 9.53±0.57 | 1.13±0.19^b^ | 196.38±8.02^b^ | 19.07±0.95 | 1.82±0.04 | 21.74±0.49 | 11.94±0.12 | 7.25±1.42^b^ | 0.11±0.02^b^ |
| 202N | 6.12±0.05^b^ | 23.00±1.51 | 9.62±0.53 | 2.77±0.39^a^ | 186.07±5.23^b^ | 20.29±1.83 | 1.87±0.05 | 21.97±0.58 | 11.78±0.09 | 20.56±7.43^a^ | 0.17±0.02^a^ |
| SG | 6.32±0.05^a^ | 23.30±1.31 | 9.52±0.49 | 1.71±0.24 | 195.32±6.71 | 20.28±1.49 | 1.83±0.04 | 21.60±0.41 | 11.79±0.12 | 9.23±1.57 | 0.14±0.02^a^ |
| BB | 6.19±0.03^b^ | 23.14±1.29 | 9.51±0.54 | 1.30±0.33 | 198.11±5.77 | 18.03±0.68 | 1.78±0.04 | 21.46±0.47 | 12.06±0.08 | 14.54±5.11 | 0.1±0.02^b^ |
| 0N-SG | 6.48±0.09 | 22.23±2.72^b^ | 10.03±1.75 | 0.48±0.17 | 201.67±13.80 | 17.34±1.71 | 1.74±0.06 | 21.49±0.81 | 12.36±0.36^a^ | 7.28±1.77 | 0.08±0.02 |
| 67N-SG | 6.35±0.05 | 23.77±2.26^ab^ | 9.38±0.51 | 1.39±0.16 | 194.43±13.99 | 19.97±1.52 | 1.83±0.05 | 21.37±0.54 | 11.67±0.10^b^ | 7.49±2.19 | 0.12±0.02 |
| 202N-SG | 6.19±0.08 | 23.56±2.20^ab^ | 9.32±0.54 | 2.86±0.32 | 191.98±8.07 | 22.55±3.48 | 1.90±0.07 | 21.90±0.84 | 11.53±0.09^b^ | 12.26±3.29 | 0.2±0.03 |
| 0N-BB | 6.27±0.03 | 23.96±2.13^a^ | 8.93±0.87 | 0.34±0.25 | 215.85±11.24 | 17.86±1.51 | 1.70±0.06 | 20.22±0.69 | 11.94±0.12^ab^ | 7.74±2.28 | 0.05±0.01 |
| 67N-BB | 6.23±0.06 | 23.02±2.57^ab^ | 9.69±1.05 | 0.88±0.33 | 198.33±8.76 | 18.18±1.14 | 1.81±0.07 | 22.12±0.82 | 12.21±0.18^ab^ | 7.01±1.94 | 0.09±0.03 |
| 202N-BB | 6.06±0.04 | 22.43±2.20^b^ | 9.92±0.95 | 2.68±0.73 | 180.16±6.50 | 18.04±0.93 | 1.83±0.07 | 22.04±0.85 | 12.02±0.11^ab^ | 28.87±14.38 | 0.15±0.03 |
| Apr-0N | 6.35±0.10 | 24.77±0.52 | 12.29±1.26^a^ | 0.45±0.17 | 220.18±3.73 | 19.97±0.88 | 1.79±0.04 | 21.50±0.48 | 12.01±0.23 | 12.00±1.68^b^ | 0.04±0.01^d^ |
| Apr-67N | 6.31±0.05 | 24.22±0.30 | 10.87±0.51^ab^ | 1.20±0.21 | 204.94±6.82 | 18.24±0.69 | 1.77±0.04 | 21.10±0.71 | 11.95±0.20 | 6.99±0.99^b^ | 0.06±0.01^d^ |
| Apr-202N | 6.16±0.07 | 24.44±0.54 | 9.54±0.38^bc^ | 2.70±0.52 | 188.30±3.30 | 18.00±1.21 | 1.90±0.07 | 22.58±0.89 | 11.87±0.18 | 10.52±1.64^b^ | 0.08±0.01^cd^ |
| Jun-0N | 6.43±0.09 | 29.68±0.74 | 9.36±1.14^bcd^ | 0.67±0.44 | 169.15±6.71 | 15.21±1.12 | 1.60±0.06 | 19.68±1.00 | 12.31±0.43 | 9.25±2.30^b^ | 0.04±0.01^d^ |
| Jun-67N | 6.38±0.09 | 31.13±0.76 | 10.43±1.10^ab^ | 1.59±0.39 | 159.77±9.47 | 16.23±1.30 | 1.75±0.07 | 21.16±1.06 | 12.05±0.15 | 13.09±2.54^b^ | 0.1±0.01^cd^ |
| Jun-202N | 6.12±0.12 | 29.60±0.85 | 11.71±0.84^a^ | 3.90±0.76 | 163.52±4.73 | 16.88±1.04 | 1.90±0.11 | 22.43±1.30 | 11.82±0.16 | 49.38±17.44^a^ | 0.17±0.03^b^ |
| Aug-0N | 6.30±0.04 | 15.35±0.74 | 6.46±0.62^e^ | 0.06±0.04 | 241.21±5.54 | 17.78±2.79 | 1.75±0.10 | 21.01±1.16 | 12.00±0.18 | 1.41±0.43^b^ | 0.11±0.01^bc^ |
| Aug-67N | 6.19±0.06 | 14.83±0.87 | 7.30±0.57^de^ | 0.61±0.27 | 224.43±8.92 | 22.76±1.58 | 1.95±0.08 | 22.96±0.57 | 11.83±0.28 | 1.68±0.36^b^ | 0.17±0.03^b^ |
| Aug-202N | 6.08±0.03 | 14.95±0.53 | 7.61±0.61^cde^ | 1.71±0.47 | 206.38±7.76 | 26.01±4.66 | 1.80±0.06 | 20.89±0.77 | 11.64±0.14 | 1.80±0.12^b^ | 0.27±0.02^a^ |
| Apr-SG | 6.34±0.08 | 24.69±0.36 | 11.30±0.90 | 1.95±0.42^ab^ | 202.78±4.65 | 19.57±0.72 | 1.89±0.05 | 22.14±0.64 | 11.75±0.15 | 8.78±1.17 | 0.08±0.01 |
| Apr-BB | 6.20±0.04 | 24.26±0.36 | 10.39±0.46 | 1.12±0.40^bc^ | 204.24±7.05 | 17.86±0.79 | 1.76±0.03 | 21.38±0.60 | 12.11±0.15 | 10.53±1.45 | 0.04±0.01 |
| Jun-SG | 6.43±0.10 | 30.00±0.73 | 9.69±0.48 | 1.76±0.44^ab^ | 158.05±6.59 | 16.06±0.88 | 1.72±0.07 | 20.55±0.87 | 11.95±0.29 | 17.08±2.46 | 0.13±0.03 |
| Jun-BB | 6.19±0.07 | 30.31±0.63 | 11.35±1.01 | 2.47±0.78^a^ | 169.01±4.65 | 16.24±1.01 | 1.79±0.08 | 21.73±1.03 | 12.13±0.11 | 31.60±13.87 | 0.09±0.02 |
| Aug-SG | 6.20±0.05 | 15.23±0.49 | 7.57±0.57 | 1.42±0.42^b^ | 225.13±6.71 | 25.20±3.81 | 1.89±0.04 | 22.10±0.53 | 11.67±0.17 | 1.82±0.20 | 0.21±0.03 |
| Aug-BB | 6.17±0.04 | 14.84±0.64 | 6.80±0.40 | 0.31±0.15^c^ | 221.09±8.77 | 19.98±1.39 | 1.79±0.08 | 21.26±0.85 | 11.93±0.17 | 1.49±0.28 | 0.16±0.03 |
| Apr-0N-SG | 6.49±0.20 | 24.06±0.83 | 14.44±2.00 | 0.80±0.14 | 211.52±1.68 | 20.59±0.59 | 1.79±0.07 | 21.43±0.07 | 12.00±0.45 | 9.50±1.58^bc^ | 0.06±0.02 |
| Apr-67N-SG | 6.36±0.08 | 24.22±0.49 | 10.54±0.89 | 1.54±0.24 | 204.90±11.62 | 18.91±1.00 | 1.80±0.01 | 20.96±0.38 | 11.67±0.23 | 5.74±0.82^bc^ | 0.07±0 |
| Apr-202N-SG | 6.23±0.15 | 25.57±0.17 | 9.98±0.61 | 3.13±0.62 | 194.83±2.38 | 19.55±1.75 | 2.04±0.08 | 23.79±1.19 | 11.65±0.13 | 11.34±1.68^bc^ | 0.09±0.02 |
| Apr-0N-BB | 6.25±0.06 | 25.25±0.53 | 10.86±0.84 | 0.21±0.14 | 225.96±1.84 | 19.56±1.49 | 1.79±0.06 | 21.55±0.87 | 12.01±0.26 | 13.66±2.16^bc^ | 0.02±0.01 |
| Apr-67N-BB | 6.26±0.06 | 24.22±0.46 | 11.21±0.62 | 0.87±0.21 | 204.99±9.87 | 17.57±0.97 | 1.73±0.09 | 21.23±1.54 | 12.22±0.28 | 8.23±1.62^bc^ | 0.04±0.01 |
| Apr-202N-BB | 6.09±0.03 | 23.31±0.34 | 9.10±0.37 | 2.27±0.88 | 181.77±2.51 | 16.45±1.36 | 1.77±0.04 | 21.38±1.04 | 12.09±0.31 | 9.69±3.17^bc^ | 0.07±0.02 |
| Jun-0N-SG | 6.58±0.17 | 28.52±0.57 | 9.49±1.55 | 0.52±0.34 | 160.41±4.48 | 15.63±0.08 | 1.58±0.04 | 20.19±2.00 | 12.74±0.94 | 10.01±2.00^bc^ | 0.05±0 |
| Jun-67N-SG | 6.50±0.05 | 31.21±1.14 | 9.51±0.82 | 1.49±0.35 | 150.67±17.47 | 16.25±2.58 | 1.71±0.08 | 20.12±0.86 | 11.80±0.07 | 15.09±3.04^bc^ | 0.12±0 |
| Jun-202N-SG | 6.25±0.21 | 29.76±1.40 | 10.00±0.43 | 2.87±0.69 | 163.85±6.69 | 16.18±0.65 | 1.83±0.17 | 21.23±1.97 | 11.59±0.24 | 23.77±2.08^b^ | 0.2±0.05 |
| Jun-0N-BB | 6.33±0.05 | 30.45±0.96 | 9.28±1.77 | 0.77±0.75 | 174.97±9.88 | 14.93±2.03 | 1.61±0.10 | 19.34±1.08 | 12.03±0.25 | 8.74±3.92^bc^ | 0.04±0.02 |
| Jun-67N-BB | 6.25±0.14 | 31.05±1.25 | 11.35±2.13 | 1.70±0.78 | 168.87±7.78 | 16.21±1.34 | 1.80±0.14 | 22.21±1.94 | 12.31±0.20 | 11.09±4.35^bc^ | 0.08±0 |
| Jun-202N-BB | 5.99±0.11 | 29.44±1.27 | 13.43±0.62 | 4.93±1.16 | 163.18±8.19 | 17.58±2.13 | 1.96±0.16 | 23.64±1.77 | 12.05±0.13 | 74.98±29.33^a^ | 0.14±0.05 |
| Aug-0N-SG | 6.39±0.04 | 14.10±0.16 | 6.15±0.97 | 0.11±0.08 | 233.07±4.94 | 15.80±4.28 | 1.86±0.05 | 22.87±0.40 | 12.33±0.14 | 2.32±0.44^bc^ | 0.13±0 |
| Aug-67N-SG | 6.19±0.05 | 15.87±0.79 | 8.09±0.41 | 1.14±0.27 | 227.73±18.46 | 24.75±1.08 | 1.99±0.06 | 23.02±0.66 | 11.55±0.20 | 1.64±0.35^c^ | 0.18±0.02 |
| Aug-202N-SG | 6.09±0.06 | 15.35±0.96 | 7.99±1.25 | 2.56±0.53 | 217.25±3.50 | 31.92±8.56 | 1.82±0.06 | 20.67±0.68 | 11.36±0.09 | 1.66±0.17^c^ | 0.3±0.02 |
| Aug-0N-BB | 6.24±0.03 | 16.19±0.98 | 6.66±0.87 | 0.02±0.00 | 246.63±7.31 | 19.10±3.83 | 1.68±0.16 | 19.78±1.57 | 11.78±0.18 | 0.80±0.24^c^ | 0.09±0.01 |
| Aug-67N-BB | 6.18±0.12 | 13.79±1.44 | 6.51±0.92 | 0.07±0.05 | 221.13±6.81 | 20.76±2.72 | 1.91±0.16 | 22.91±1.09 | 12.10±0.52 | 1.73±0.71^c^ | 0.16±0.06 |
| Aug-202N-BB | 6.08±0.04 | 14.55±0.58 | 7.22±0.41 | 0.85±0.25 | 195.52±13.07 | 20.10±0.65 | 1.77±0.13 | 21.10±1.56 | 11.92±0.12 | 1.94±0.14^c^ | 0.23±0.03 |

^†^ G, grass green up; H1, initial grass harvest; H2, second grass harvest; 0N, no N fertilization; 67N, 67 kg N ha^-1^ fertilization; 202N, 202 kg N ha^-1^ fertilization; SG, switchgrass; BB, big bluestem.

^‡^SWC, soil water content; DOC, dissolved organic C; DON, dissolved organic N; NP, nitrification potential.

Hu, J., Richwine, J. D., Keyser, P. D., Li, L., Yao, F., Jagadamma, S., & DeBruyn, J. M. (2021b). Ammonia-oxidizing bacterial communities are affected by nitrogen fertilization and grass species in native C4 grassland soils. *PeerJ*, *9*. https://doi.org/10.7717/peerj.12592
