# Supplementary material for: Urea fertilization and grass species alter microbial nitrogen cycling capacity and activity in a C4 native grassland
Source: PeerJ. 2022 Aug 12;10:e13874. doi: 10.7717/peerj.13874 (PMC9377331; doi:10.7717/peerj.13874)
Supplement: Supplemental Information 4 — F-values are reported. Significant models are indicated by asterisks. From Hu et al. (2021b). [file peerj-10-13874-s004.docx]

**Table S2.** **Results of mixed model ANOVA (based on GLIMMIX procedure in SAS) testing effects of sampling time (season), nitrogen fertilization and grass species on soil properties.** F-values are reported. Significant models are indicated by asterisks. From Hu et al., 2021b.

| Effect | pH | SWC^†^ | NH_4_^+^-N | NO_3_^-^-N | DOC | DON | Total C | Total N | C: N ratio | N_2_O-N | Nitrification  potential |
| --- | --- | --- | --- | --- | --- | --- | --- | --- | --- | --- | --- |
| Season (S) | 3.26 | 388.39*** | 23.06*** | 9.00*** | 57.99*** | 6.10** | 0.43 | 1.11 | 0.98 | 12.46*** | 31.13*** |
| Nitrogen (N) | 11.70*** | 0.31 | 0.01 | 31.14*** | 7.71** | 1.20 | 0.88 | 2.44 | 2.15 | 5.94** | 21.85*** |
| Grass (G) | 11.53** | 0.01 | 0.04 | 1.95 | 0.20 | 1.91 | 0.11 | 0.82 | 2.01 | 2.25 | 10.44** |
| N×G | 0.06 | 3.75* | 1.09 | 0.17 | 2.62 | 1.06 | 1.13 | 0.08 | 4.35* | 2.38 | 0.28 |
| S×N | 0.59 | 0.89 | 3.40* | 1.41 | 1.48 | 1.64 | 1.58 | 1.79 | 0.24 | 5.36** | 2.72* |
| S×G | 1.99 | 0.35 | 3.05 | 5.42** | 0.98 | 0.83 | 1.22 | 1.52 | 0.24 | 1.78 | 0.13 |
| S×N×G | 0.13 | 0.57 | 1.35 | 1.61 | 0.21 | 1.13 | 0.84 | 0.53 | 0.26 | 3.00* | 0.30 |

Significance level: * *p*-value ≤ 0.05; ** *p*-value ≤ 0.01; *** *p*-value ≤ 0.001.

^†^SWC, soil water content; DOC, dissolved organic C; DON, dissolved organic N.

Hu, J., Richwine, J. D., Keyser, P. D., Li, L., Yao, F., Jagadamma, S., & DeBruyn, J. M. (2021b). Ammonia-oxidizing bacterial communities are affected by nitrogen fertilization and grass species in native C4 grassland soils. *PeerJ*, *9*. https://doi.org/10.7717/peerj.12592
