# Supplementary material for: Urea fertilization and grass species alter microbial nitrogen cycling capacity and activity in a C4 native grassland
Source: PeerJ. 2022 Aug 12;10:e13874. doi: 10.7717/peerj.13874 (PMC9377331; doi:10.7717/peerj.13874)
Supplement: Supplemental Information 5 — Different lowercase letters indicate significant differences between treatment levels within groups. The letters were only shown in groups that have significant effects. Means were compared using Least Significant Difference (LSD) tests (α = 0.05). nifH data is from Hu et al. (2021c). amoA data is from Hu et al. (2021b). [file peerj-10-13874-s005.docx]

**Table S3. Absolute abundances of *nifH*, *amoA*, *nirK*, *nirS*, *nosZ*, and 16S rRNA genes as well as relative abundances of *nifH*, *amoA*, *nirK*, *nirS*, and *nosZ* genes in relation to agricultural season, nitrogen fertilization, grass species, and their combinations.** Different lowercase letters indicate significant differences between treatment levels within groups. The letters were only shown in groups that have significant effects. Means were compared using Least Significant Difference (LSD) tests (α = 0.05). *nifH* data is from Hu et al., 2021c. *amoA* data is from Hu et al., 2021b.

| Factor^†^ | *nifH* | | *amoA* | *nirK* | *nirS* | *nosZ* | 16S rRNA | *nifH* | *amoA* | *nirK* | *nirS* | | *nosZ* |
| --- | --- | --- | --- | --- | --- | --- | --- | --- | --- | --- | --- | --- | --- |
|  | Log(gene copies g^-1^ dry weight soil) | | | | | | | Log(functional gene copies/16S rRNA gene copies) | | | | | |
| April (G) | 6.61±0.07^b^ | 6.01±0.14^b^ | | 7.21±0.07^b^ | 7.27±0.06^b^ | 7.30±0.06^b^ | 9.65±0.05^b^ | -3.05±0.04^b^ | -3.65±0.10^b^ | -2.44±0.03^b^ | -2.39±0.04^b^ | -2.35±0.02^a^ | |
| June (H1) | 6.17±0.06^c^ | 5.27±0.10^c^ | | 6.93±0.05^c^ | 7.12±0.04^c^ | 7.00±0.04^c^ | 9.53±0.04^c^ | -3.36±0.05^c^ | -4.26±0.10^c^ | -2.59±0.03^c^ | -2.41±0.04^b^ | -2.52±0.03^b^ | |
| August (H2) | 7.04±0.04^a^ | 6.93±0.09^a^ | | 7.59±0.03^a^ | 7.74±0.04^a^ | 7.55±0.03^a^ | 9.86±0.03^a^ | -2.82±0.04^a^ | -2.93±0.10^a^ | -2.27±0.04^a^ | -2.12±0.03^a^ | -2.31±0.03^a^ | |
| 0N | 6.65±0.10 | 5.68±0.20^b^ | | 7.23±0.09 | 7.38±0.08 | 7.29±0.07 | 9.69±0.05 | -3.05±0.08 | -4.01±0.17^b^ | -2.46±0.05 | -2.31±0.05 | -2.41±0.04 | |
| 67N | 6.57±0.11 | 6.17±0.18^a^ | | 7.25±0.10 | 7.41±0.09 | 7.28±0.07 | 9.69±0.06 | -3.12±0.06 | -3.51±0.13^a^ | -2.44±0.05 | -2.28±0.05 | -2.40±0.03 | |
| 202N | 6.60±0.09 | 6.35±0.18^a^ | | 7.25±0.07 | 7.33±0.07 | 7.29±0.07 | 9.66±0.05 | -3.06±0.07 | -3.31±0.15^a^ | -2.40±0.03 | -2.33±0.05 | -2.37±0.03 | |
| SG | 6.69±0.08 | 6.21±0.16^a^ | | 7.32±0.06^a^ | 7.46±0.06^a^ | 7.32±0.06 | 9.73±0.04 | -3.03±0.05 | -3.51±0.13^a^ | -2.41±0.04 | -2.26±0.04^a^ | -2.40±0.03 | |
| BB | 6.52±0.09 | 5.92±0.16^b^ | | 7.17±0.07^b^ | 7.29±0.07^b^ | 7.25±0.06 | 9.63±0.04 | -3.11±0.06 | -3.71±0.14^b^ | -2.46±0.04 | -2.35±0.03^b^ | -2.38±0.02 | |
| 0N-SG | 6.67±0.15 | 5.90±0.29 | | 7.26±0.12 | 7.38±0.10^b^ | 7.25±0.10 | 9.71±0.07 | -3.03±0.12 | -3.81±0.25 | -2.44±0.08 | -2.33±0.08 | -2.46±0.07 | |
| 67N-SG | 6.74±0.13 | 6.35±0.25 | | 7.43±0.10 | 7.62±0.10^a^ | 7.40±0.10 | 9.80±0.08 | -3.06±0.07 | -3.45±0.19 | -2.37±0.06 | -2.18±0.05 | -2.40±0.05 | |
| 202N-SG | 6.67±0.13 | 6.39±0.27 | | 7.26±0.11 | 7.38±0.10^b^ | 7.32±0.10 | 9.67±0.07 | -3.00±0.10 | -3.29±0.23 | -2.41±0.06 | -2.29±0.08 | -2.35±0.05 | |
| 0N-BB | 6.62±0.15 | 5.47±0.27 | | 7.20±0.13 | 7.39±0.12^b^ | 7.33±0.10 | 9.68±0.08 | -3.07±0.10 | -4.22±0.22 | -2.48±0.07 | -2.30±0.07 | -2.36±0.04 | |
| 67N-BB | 6.40±0.17 | 5.99±0.25 | | 7.07±0.14 | 7.19±0.12^c^ | 7.17±0.10 | 9.57±0.07 | -3.17±0.11 | -3.58±0.19 | -2.50±0.08 | -2.38±0.06 | -2.41±0.05 | |
| 202N-BB | 6.53±0.13 | 6.31±0.27 | | 7.24±0.09 | 7.28±0.10^bc^ | 7.25±0.09 | 9.64±0.07 | -3.11±0.09 | -3.33±0.21 | -2.40±0.04 | -2.36±0.05 | -2.39±0.03 | |
| Apr-0N | 6.66±0.12 | 5.72±0.26 | | 7.19±0.11 | 7.26±0.07 | 7.28±0.11 | 9.64±0.09 | -2.98±0.07 | -3.92±0.20 | -2.45±0.04 | -2.37±0.05 | -2.36±0.04 | |
| Apr-67N | 6.54±0.15 | 6.02±0.21 | | 7.15±0.16 | 7.26±0.16 | 7.26±0.11 | 9.61±0.09 | -3.07±0.08 | -3.59±0.15 | -2.46±0.08 | -2.34±0.08 | -2.35±0.04 | |
| Apr-202N | 6.62±0.09 | 6.29±0.22 | | 7.30±0.11 | 7.27±0.08 | 7.37±0.11 | 9.71±0.09 | -3.09±0.07 | -3.43±0.15 | -2.41±0.05 | -2.44±0.06 | -2.34±0.04 | |
| Jun-0N | 6.21±0.12 | 4.83±0.18 | | 6.91±0.11 | 7.12±0.07 | 7.03±0.09 | 9.59±0.10 | -3.38±0.12 | -4.76±0.15 | -2.67±0.06 | -2.47±0.09 | -2.56±0.07 | |
| Jun-67N | 6.12±0.13 | 5.45±0.09 | | 6.93±0.10 | 7.15±0.10 | 6.99±0.06 | 9.51±0.06 | -3.40±0.08 | -4.06±0.09 | -2.58±0.06 | -2.36±0.08 | -2.52±0.04 | |
| Jun-202N | 6.18±0.10 | 5.52±0.04 | | 6.95±0.04 | 7.07±0.07 | 6.98±0.03 | 9.48±0.05 | -3.29±0.08 | -3.96±0.05 | -2.52±0.04 | -2.41±0.06 | -2.50±0.03 | |
| Aug-0N | 7.07±0.07 | 6.50±0.13 | | 7.60±0.06 | 7.76±0.05 | 7.55±0.06 | 9.86±0.03 | -2.79±0.05 | -3.36±0.16 | -2.26±0.08 | -2.09±0.03 | -2.31±0.05 | |
| Aug-67N | 7.06±0.06 | 7.05±0.11 | | 7.67±0.05 | 7.81±0.08 | 7.6±0.06 | 9.94±0.06 | -2.88±0.05 | -2.89±0.09 | -2.27±0.07 | -2.14±0.06 | -2.34±0.07 | |
| Aug-202N | 7.00±0.06 | 7.24±0.03 | | 7.51±0.06 | 7.65±0.07 | 7.51±0.06 | 9.78±0.06 | -2.78±0.08 | -2.54±0.08 | -2.27±0.04 | -2.13±0.06 | -2.26±0.04 | |
| Apr-SG | 6.72±0.10 | 6.28±0.16 | | 7.34±0.09 | 7.39±0.08 | 7.41±0.09 | 9.75±0.07 | -3.03±0.05 | -3.48±0.12 | -2.42±0.05 | -2.36±0.05 | -2.34±0.04 | |
| Apr-BB | 6.49±0.08 | 5.74±0.19 | | 7.09±0.09 | 7.14±0.07 | 7.19±0.07 | 9.55±0.05 | -3.06±0.07 | -3.81±0.16 | -2.46±0.05 | -2.41±0.05 | -2.36±0.02 | |
| Jun-SG | 6.27±0.07 | 5.33±0.14 | | 7.00±0.04 | 7.20±0.05 | 7.00±0.04 | 9.55±0.05 | -3.28±0.09 | -4.22±0.14 | -2.55±0.04 | -2.35±0.08 | -2.55±0.04 | |
| Jun-BB | 6.07±0.10 | 5.20±0.15 | | 6.87±0.09 | 7.03±0.06 | 7.00±0.06 | 9.50±0.07 | -3.43±0.05 | -4.30±0.16 | -2.63±0.05 | -2.47±0.02 | -2.50±0.03 | |
| Aug-SG | 7.09±0.04 | 7.03±0.11 | | 7.62±0.06 | 7.79±0.06 | 7.55±0.05 | 9.87±0.06 | -2.79±0.04 | -2.84±0.12 | -2.26±0.07 | -2.08±0.04 | -2.32±0.06 | |
| Aug-BB | 7.00±0.06 | 6.82±0.15 | | 7.56±0.04 | 7.69±0.05 | 7.55±0.05 | 9.85±0.04 | -2.85±0.06 | -3.02±0.17 | -2.28±0.03 | -2.16±0.04 | -2.29±0.02 | |
| Apr-0N-SG | 6.74±0.24 | 6.10±0.23 | | 7.27±0.18 | 7.31±0.14 | 7.35±0.20 | 9.71±0.17 | -2.96±0.09 | -3.60±0.09 | -2.44±0.04 | -2.40±0.04 | -2.36±0.04 | |
| Apr-67N-SG | 6.79±0.11 | 6.36±0.28 | | 7.44±0.15 | 7.57±0.12 | 7.46±0.13 | 9.79±0.09 | -3.00±0.04 | -3.43±0.24 | -2.35±0.11 | -2.22±0.07 | -2.33±0.09 | |
| Apr-202N-SG | 6.63±0.19 | 6.36±0.40 | | 7.30±0.21 | 7.30±0.16 | 7.43±0.20 | 9.76±0.15 | -3.14±0.12 | -3.40±0.30 | -2.46±0.09 | -2.47±0.10 | -2.34±0.09 | |
| Apr-0N-BB | 6.58±0.05 | 5.33±0.37 | | 7.11±0.13 | 7.22±0.07 | 7.21±0.13 | 9.57±0.09 | -2.99±0.13 | -4.24±0.29 | -2.46±0.08 | -2.35±0.09 | -2.36±0.08 | |
| Apr-67N-BB | 6.28±0.19 | 5.68±0.18 | | 6.86±0.13 | 6.95±0.13 | 7.06±0.04 | 9.43±0.04 | -3.14±0.16 | -3.75±0.15 | -2.56±0.09 | -2.47±0.09 | -2.37±0.02 | |
| Apr-202N-BB | 6.61±0.04 | 6.21±0.26 | | 7.29±0.14 | 7.24±0.06 | 7.32±0.12 | 9.66±0.11 | -3.05±0.09 | -3.45±0.15 | -2.37±0.03 | -2.42±0.08 | -2.34±0.02 | |
| Jun-0N-SG | 6.22±0.14 | 4.91±0.29 | | 6.92±0.03 | 7.11±0.08 | 6.93±0.03 | 9.58±0.13 | -3.36±0.26 | -4.67±0.21 | -2.66±0.10 | -2.47±0.19 | -2.65±0.12 | |
| Jun-67N-SG | 6.28±0.10 | 5.53±0.13 | | 7.11±0.06 | 7.33±0.03 | 7.08±0.09 | 9.56±0.08 | -3.28±0.10 | -4.03±0.19 | -2.45±0.04 | -2.23±0.10 | -2.48±0.02 | |
| Jun-202N-SG | 6.32±0.18 | 5.56±0.06 | | 6.96±0.09 | 7.16±0.10 | 7.01±0.07 | 9.52±0.09 | -3.20±0.15 | -3.96±0.07 | -2.56±0.03 | -2.36±0.11 | -2.51±0.03 | |
| Jun-0N-BB | 6.20±0.24 | 4.76±0.28 | | 6.90±0.25 | 7.13±0.14 | 7.14±0.18 | 9.59±0.18 | -3.40±0.10 | -4.84±0.24 | -2.69±0.08 | -2.46±0.04 | -2.46±0.03 | |
| Jun-67N-BB | 5.96±0.21 | 5.38±0.15 | | 6.75±0.12 | 6.98±0.12 | 6.90±0.04 | 9.47±0.10 | -3.51±0.11 | -4.09±0.08 | -2.72±0.02 | -2.49±0.05 | -2.57±0.07 | |
| Jun-202N-BB | 6.05±0.04 | 5.47±0.05 | | 6.95±0.02 | 6.98±0.07 | 6.96±0.01 | 9.44±0.05 | -3.39±0.02 | -3.96±0.10 | -2.49±0.07 | -2.46±0.02 | -2.48±0.05 | |
| Aug-0N-SG | 7.06±0.09 | 6.68±0.20 | | 7.59±0.13 | 7.72±0.05 | 7.47±0.08 | 9.83±0.04 | -2.77±0.07 | -3.15±0.24 | -2.24±0.16 | -2.11±0.01 | -2.36±0.11 | |
| Aug-67N-SG | 7.15±0.01 | 7.17±0.02 | | 7.73±0.09 | 7.97±0.07 | 7.66±0.08 | 10.06±0.06 | -2.91±0.06 | -2.88±0.07 | -2.33±0.15 | -2.09±0.12 | -2.40±0.14 | |
| Aug-202N-SG | 7.05±0.08 | 7.24±0.02 | | 7.53±0.10 | 7.69±0.14 | 7.54±0.07 | 9.73±0.07 | -2.68±0.01 | -2.49±0.05 | -2.20±0.03 | -2.05±0.07 | -2.19±0.01 | |
| Aug-0N-BB | 7.08±0.12 | 6.31±0.12 | | 7.60±0.05 | 7.81±0.10 | 7.63±0.05 | 9.89±0.05 | -2.81±0.07 | -3.57±0.16 | -2.28±0.07 | -2.07±0.06 | -2.26±0.02 | |
| Aug-67N-BB | 6.97±0.10 | 6.92±0.21 | | 7.61±0.02 | 7.65±0.03 | 7.54±0.07 | 9.83±0.03 | -2.86±0.09 | -2.90±0.19 | -2.22±0.01 | -2.18±0.01 | -2.29±0.04 | |
| Aug-202N-BB | 6.94±0.11 | 7.24±0.06 | | 7.48±0.09 | 7.61±0.09 | 7.49±0.11 | 9.83±0.12 | -2.89±0.16 | -2.59±0.17 | -2.34±0.06 | -2.21±0.09 | -2.34±0.04 | |

^†^ G, grass green up; H1, initial grass harvest; H2, second grass harvest; 0N, no N fertilization; 67N, 67 kg N ha^-1^ fertilization; 202N, 202 kg N ha^-1^ fertilization; SG, switchgrass; BB, big bluestem.

Hu, J., Richwine, J. D., Keyser, P. D., Li, L., Yao, F., Jagadamma, S., & DeBruyn, J. M. (2021b). Ammonia-oxidizing bacterial communities are affected by nitrogen fertilization and grass species in native C4 grassland soils. *PeerJ*, *9*. https://doi.org/10.7717/peerj.12592

Hu, J., Richwine, J. D., Keyser, P. D., Li, L., Yao, F., Jagadamma, S., & DeBruyn, J. M. (2021c). Nitrogen fertilization and native C_4_ grass species alter abundance, activity, and diversity of soil diazotrophic communities. *Frontiers in Microbiology*, *12*, 675693. https://doi.org/10.3389/fmicb.2021.675693
